# Crystal Structure of the *S. solfataricus* Archaeal Exosome Reveals Conformational Flexibility in the RNA-Binding Ring

**DOI:** 10.1371/journal.pone.0008739

**Published:** 2010-01-15

**Authors:** Changrui Lu, Fang Ding, Ailong Ke

**Affiliations:** Department of Molecular Biology and Genetics, Cornell University, Ithaca, New York, United States of America; University of Oulu, Finland

## Abstract

**Background:**

The exosome complex is an essential RNA 3′-end processing and degradation machinery. In archaeal organisms, the exosome consists of a catalytic ring and an RNA-binding ring, both of which were previously reported to assume three-fold symmetry.

**Methodology/Principal Findings:**

Here we report an asymmetric 2.9 Å *Sulfolobus solfataricus* archaeal exosome structure in which the three-fold symmetry is broken due to combined rigid body and thermal motions mainly within the RNA-binding ring. Since increased conformational flexibility was also observed in the RNA-binding ring of the related bacterial PNPase, we speculate that this may reflect an evolutionarily conserved mechanism to accommodate diverse RNA substrates for degradation.

**Conclusion/Significance:**

This study clearly shows the dynamic structures within the RNA-binding domains, which provides additional insights on mechanism of asymmetric RNA binding and processing.

## Introduction

The exosome and its related bacterial polynucleotide phosphorylase (PNPase) represent a class of conserved multi-subunit protein complexes responsible for the 3′-to-5′ processing and degradation of RNA [1], [2], [3]. In the nucleus of eukaryotic cells, the exosome is responsible for the 3′-end trimming of rRNA, snRNA, and snoRNA, but also plays a major role in the degradation of the spliced introns and pre-mRNAs that fail the quality control processes [4], [5], [6], [7], [8], [9], [10], [11], [12], [13]. In addition, the nuclear exosome participates in nuclear surveillance pathways to degrade aberrant rRNA, tRNA, and mRNA transcripts [6], [11], [12], [14], [15], [16], [17]. In the cytoplasm, the exosome is a key player during mRNA turnover, and is responsible for the 3′-to-5′ degradation of normal mRNA after deadenylation as well as for the products of endonucleolytic events [18], including those of the RNAi pathway [19]. Like the nuclear exosome, the cytoplasmic exosome also participates in mRNA surveillance pathways such as nonsense-mediated decay, non-stop decay, no-go decay, and ARE-mediated decay to eliminate aberrant mRNAs [20], [21], [22], [23], [24].

The function and architecture of the exosome is conserved among all three kingdoms of life. Its bacterial counterpart, PNPase, is an 81 KDa polypeptide that trimerizes to form a ∼240 KDa complex. The primary sequence of the PNPase contains two domains homologous to RNase PH, followed by RNA-binding KH and S1 domains [25]. Architecturally, the ∼250 KDa prototypic archaeal exosome is similar to the bacterial PNPase, consisting of a catalytic ring containing a trimer of Rrp41/Rrp42 heterodimers, and with a trimetric Rrp4 or Csl4 RNA-binding ring stacked on the top. Both Rrp41 and Rrp42 are homologous to RNase PH, but only Rrp41 possesses the phosphorolytic 3′-to-5′ exoribonuclease activity [26]. The core eukaryotic exosome consists of nine different subunits [27]. Six of them classify into two RNase PH-like groups: Rrp41-like (Rrp41, Rrp46, and Mtr3) and Rrp42-like (Rrp42, Rrp43, and Rrp45), however, in yeast and human none of these subunits possess any exoribonuclease activity [27], [28]. The rest of the three subunits (Rrp4, Rrp40, and Csl4) are predicted to be RNA binding proteins [10]. The eukaryotic core exosome interacts with many protein cofactors including 3′-to-5′ exoribonuclease, RNA helicase, and polyadenylase to carry out its RNA processing and degradation functions. Crystal structures of the PNPase [25], [29], archaeal [30], [31], [32], and eukaryotic [27] exosomes have been determined, from which it has become clear that these complexes adopt a very similar doughnut-shaped architecture.

The structure and enzymatic mechanism of the archaeal exosome have been extensively studied. Published structural work revealed that the archaeal exosomes adopt a three-fold symmetric structure, where a trimeric RNA-binding ring packs on top of the catalytic ring consisting of a trimer of Rrp41/Rrp42 heterodimers. A large “processing chamber” was found inside the catalytic ring, where three identical phosphorolytic active sites reside. RNA substrate likely gains access to the “processing chamber” from the side of the RNA-binding ring through a narrow pore at the neck region, which only allows the passage of a single-stranded (ss) RNA [31], [32], [33]}. The proposed recruitment pathway is consistent with the observation that the archaeal exosome is stalled by strong RNA secondary structures, leaving RNA products with 7–9 nucleotides-long 3′ overhangs [26], [34].

To further understand the RNA recruitment mechanism of the exosome family of macromolecular machines, we determined the crystal structure of the archaeal *Sulfolobus solfataricus* exosome. We observed that while the catalytic ring of the *S. solfataricus* exosome adopts a three-fold symmetric structure, its RNA-binding ring displays considerable rigid body and thermal motions, which broke its internal symmetry. Since conformational flexibility was also observed in the RNA-binding ring of the bacterial PNPase [25], [29], we propose that the conformational dynamics may be harnessed by the archaeal exosome, and perhaps by all exosome family members, to accommodate the binding of various RNA substrates and to disrupt their structures.

## Results

### Reconstitution and Phosphorolytic RNase Activity in the Four-Subunit Archaeal Exosome

**Figure 1:**
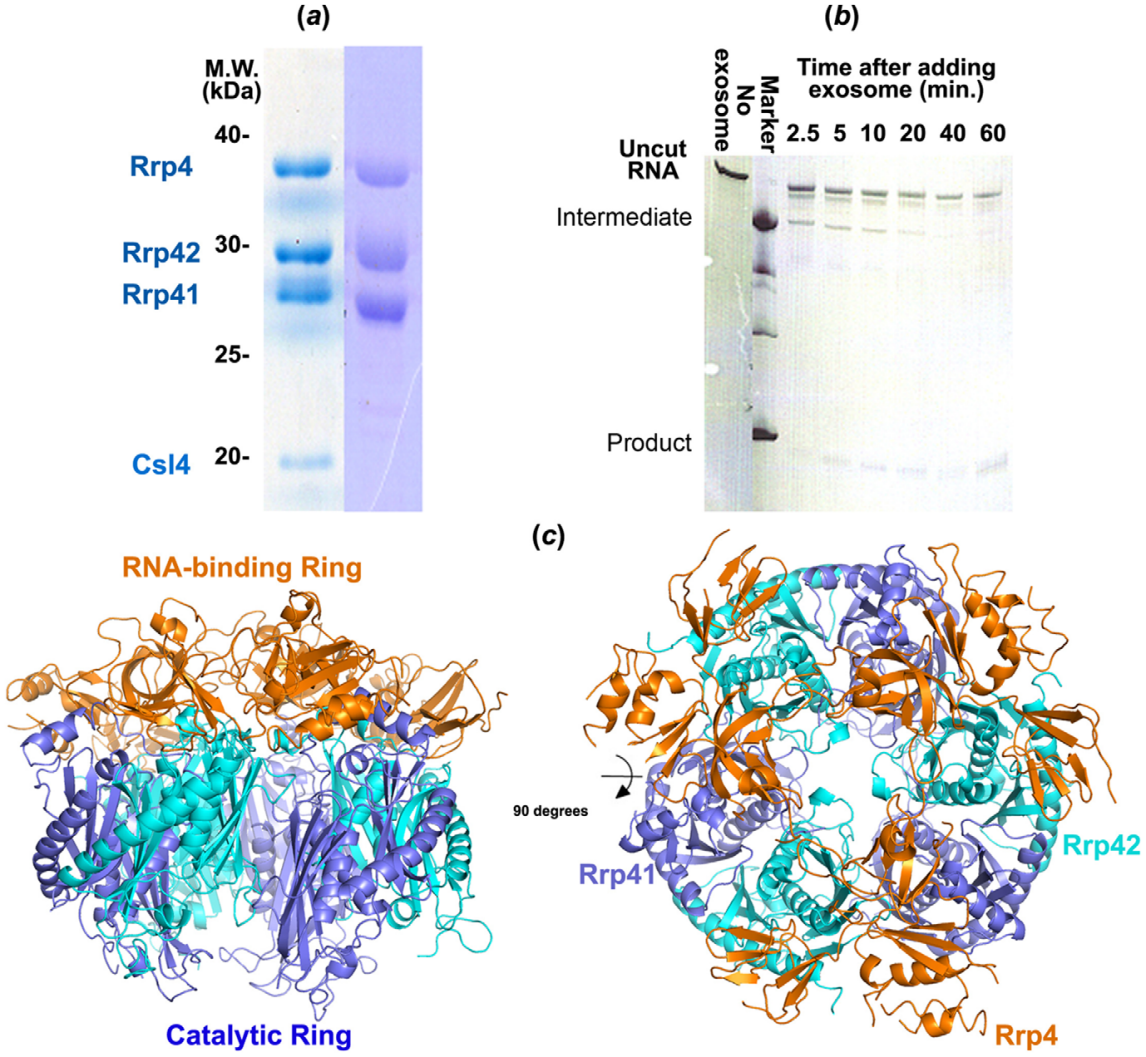
Purification and activity assay of the purified *S. solfataricus* full exosome. (*a*) SDS-PAGE of the purified *S. solfataricus* full exosome (left) and the Rrp4-exosome isoform (right). (b) RNase activity assay for the intact *S. solfataricus* exosome. The exosome is stalled by the HDV ribozyme sequence. (*c*) Side and top-down view of the 2.9 Å *S. solfataricus* exosome structure. Gold, trimeric Rrp4 RNA-binding ring; blue/cyan, Rrp41/Rrp42 catalytic ring.

When we started the work, studies had shown that all four eukaryotic exosome homologous proteins, namely Rrp41, Rrp42, Rrp4, and Csl4, are present in the purified archaeal exosome complex [31], [34], [35], [36], [37], [38]. We therefore attempted to reconstitute the archaeal *Sulfolobus solfataricus* exosome by co-expressing all four exosome subunits from a polycistronic construct in *E. coli* [39], [40], [41]. As shown in the results, while Rrp4, Rrp41, and Rrp42 were present at stoichiometric amounts in the purified complex, the Csl4 protein appeared sub-stoichiometric (Fig. 1a). The purified exosome complex was shown to be monodispersed and homogeneous under negative staining EM (data not shown).

To demonstrate the phosphorylytic RNase activity in our purified archaeal exosome, we incubated the purified exosome with a 254-nt RNA substrate flanked by a strong 5′-tertiary RNA structure (the hepatitis delta virus ribozyme) and a 161-nt flexible 3′ tail under multiple turnover conditions. As shown in Figure 1b, the archaeal exosome is highly processive in degrading the RNA substrate. The strong tertiary structure in the HDV ribozyme effectively blocks the action of the exosome, resulting in accumulation of a procession intermediate. The enzymatic activity is strictly phosphate-dependent as the activity decreased to background level in the absence of PO_4_^3−^ or in the presence of 20 mM SO_4_^2−^ (data not shown).

We obtained the crystals of the four-protein component archaeal exosome complex grown in ammonium sulfate and polyethylene glycol 4000 (PEG 4000). The crystals diffracted X-rays to 2.4 Å resolution at a synchrotron radiation source. Data processing by HKL2000 [42], MOSFLM [43] was problematic as a large fraction of data were rejected in all possible space groups (the most likely being P2/P21). We speculate this is due to epitaxial twinning of Rrp4-exosome and Csl4-exosome crystal lattices. Not surprisingly, no structure solution was obtained from the four-subunit *Sulfolobus solfataricus* exosome crystals.

### Crystallization and Structure Determination of the Rrp4-Exosome Isoform

We reasoned that the problematic diffraction pattern from the four-subunit exosome crystals was due to the presence of a sub-stoichiometric amount of Csl4 protein. To generate homogeneous exosome samples, the Csl4 gene was deleted from the co-expression cassette and biochemically, the resulting Rrp4-exosome isoform behaved similarly as the four-subunit complex. We independently crystallized the complex under high PEG conditions similar to that reported by the Conti group [30], [44]. A complete data set was collected to 2.9 Å showing that the crystals belong to C2 space group (Table 1).

**Table 1:** Crystallographic statistics.

Resolution (Å)	20–2.9
Space Group	C2
Unit Cell Dimensions	
*a* (Å)	151.1
*b* (Å)	145.5
*c* (Å)	97.2
α (°)	90
β (°)	93.8
γ (°)	90
Completeness (%)	99.1(95.2)
Beamline	CHESS A1
Redundancy	4.0
I/s	10.1(3.0)
R_sym_ (%)	7.8(31.5)
R_free_/R_work_	28.9/26.7
r.m.s.d. bonds (Å)	0.007
r.m.s.d. angles (°)	1.2
Average B (Å^2^)	32.9
Coordinate error (Å)	0.38

We solved the *S. solfataricus* exosome Rrp4-isoform structure by molecular replacement using the Rrp41/Rrp42 heterodimer in the *S. solfataricus* exosome catalytic core (PDB code: 2BR2) as the search model [34]. A single exosome core complex was reconstructed in the asymmetric unit after the rotation and translation searches successfully located each of the three heterodimers. Although no model corresponding to the Rrp4 protein was included in the search model, the resulting molecular replacement phases allowed the initial tracing of Rrp4 protein backbone. Despite similarities in the crystallization condition, unit cell dimensions and packing arrangement between our crystal structure and that by the Conti group, we noticed early on in our refinement that applying strict three-fold non-crystallographic symmetry (NCS), as the Conti group did [30], [34], [44], caused considerable fragmentation of the electron densities in the RNA-binding ring where the trimeric Rrp4 proteins are located. We therefore continued structure refinement without the three-fold NCS, re-traced one of the three copies of Rrp4, and continued refinement until the R_work_/R_free_ were within the acceptable range of 26.7%/28.9%.

### Conformation Flexibility Mainly Within the RNA-Binding Ring

The catalytic ring in our *S. solfataricus* Rrp4-exosome crystal structure (Fig. 1c) is very similar to that of the catalytic mutant structure from the same organism (PDB code 2JE6) previously reported [30]. The three copies of Rrp41/Rrp42 heterodimers within the catalytic ring align extremely well with their counterparts in the previous structure [30], even though the three-fold NCS was not enforced in our structure, the three heterodimers does not deviate notably from their symmetric positions. The r.m.s.d. of the Cα atom alignment is 0.6 Å for the whole catalytic ring (1529 aa) alignment, and between 0.2 to 0.4 Å for pair-wise Rrp41/Rrp45 heterodimer (501 aa) alignment within our structure. This result suggests that the positions of the heterodimers deviate slightly (on average ∼0.3 Å) from the symmetric positions.

**Figure 2:**
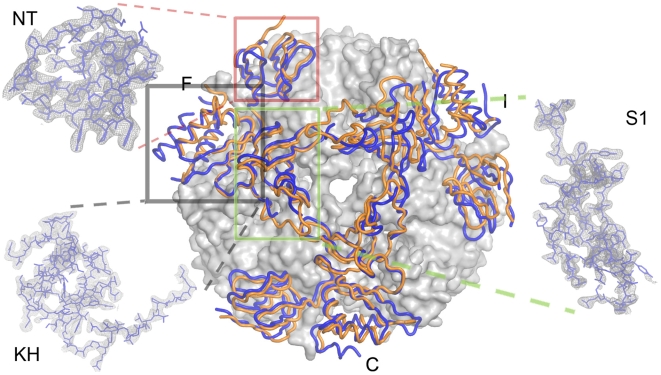
Rrp4 trimeric cap between our *S. solfataricus* exosome (blue) deviates from perfect three-fold symmetry as compared with the structure by Lorentzen *et al* [30] (orange). Subunit F and I considerably deviates (2 to 3 Å at the periphery) from Lorentzen *et al* previously report when aligning subunit C. Inlets: 2.9 Å experimental electron density map of Rrp4 subunit F contoured at 1.0 σ.

**Figure 3:**
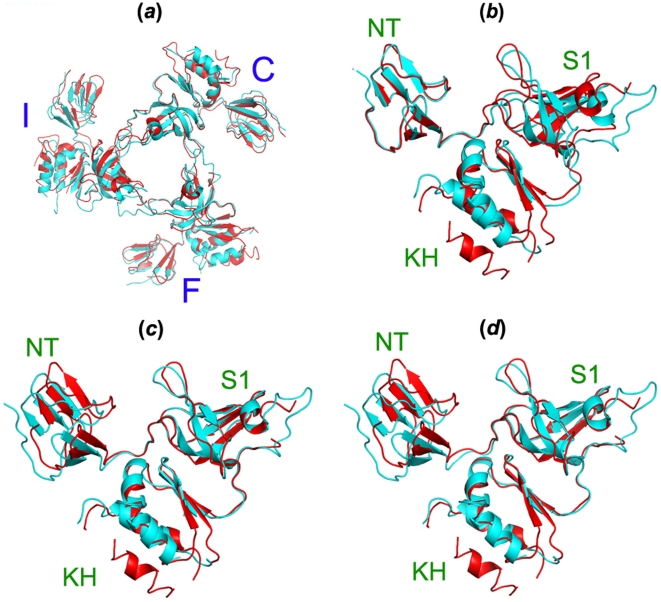
Structure alignment of Rrp4 trimeric cap between our *S. solfataricus* exosome (red) and that Lorentzen *et al* previously reported [30] (cyan). (*a*) Overall alignment of the 750− aa trimeric cap (r.m.s.d. = 1.5 Å). (*b*) Alignment of the most flexible Rrp4 subunit (Chain F) by N-ter domain, aa 1–50 (r.m.s.d. = 0.4 Å). (*c*) Alignment of Chain F subunit by S1 domain, aa 56–126 (r.m.s.d. = 0.3 Å). (*d*) Alignment of Chain F subunit by KH domain, aa 135–250 (r.m.s.d. = 0.7 Å).

The three Rrp4 subunits (designated Chains C, F, and I in our structure, respectively) in the RNA-binding ring of our structure, however, deviate significantly from their three-fold symmetric positions as reported previously (Fig. 2 [30]). When one of the Rrp4 subunits (i.e. Chain C) is superimposed with that in the Conti structure, the other two Rrp4 proteins deviate significantly from their symmetric counterparts (Fig. 2 and Fig. 3a). In addition to rigid body shifts, we observed conformational heterogeneity among Rrp4 subunits. While two of the three copies (Chain C and I) overlap reasonably well with their counterparts in the previous structure [30], with the r.m.s.d of the Cα alignment at 0.5 Å and 0.6 Å, respectively, and at 0.8 Å with each other, the third Rrp4 copy (Chain F) adopts a significantly different conformation (the r.m.s.d. of 250 Cα alignment is 1.5 Å with the previously published structure [30], Fig. 3b–d). Compared with the published structure by Lorentzen *et al*. [30], almost identical sets of intramolecular interactions were observed between the Rrp4 trimeric cap and the Rrp41/42 hetertodimers. The conformational changes are not due to changes in the crystal packing environment, as minimal crystal contacts were observed around the Rrp4 ring. The exceptionally large geometric deviation in Chain F is due to a rigid body hinge motion at the linker region between the N-terminal domain and the S1/KH domains, because when aligned separately, each domain agrees well with their counterparts in other two Rrp4 subunits (r.m.s.d. of Cα alignments for the N-ter, S1, and KH domains are at 0.3 Å (50 aa), 0.3 Å (70 aa), and 0.7 Å (115 aa), respectively, Fig. 3b–d). The hinge motion causes the S1 and KH domains in chain F to move away from the central channel and thus increases the diameter of the pore opening.

**Figure 4:**
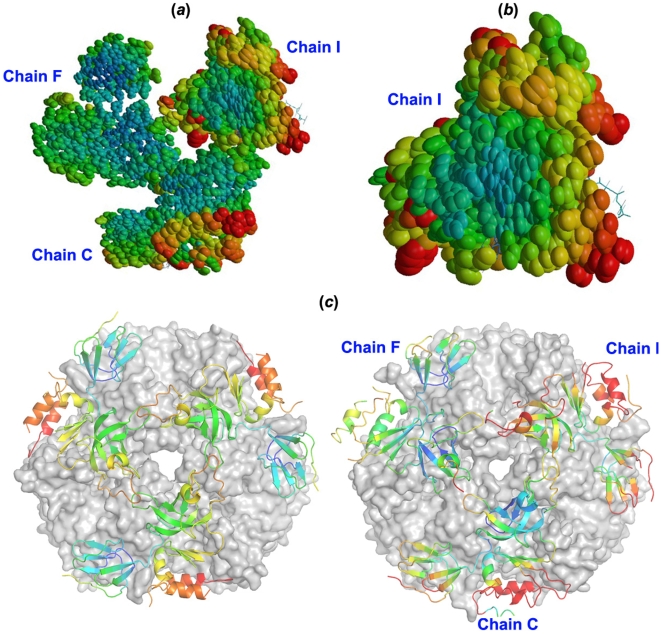
Detecting thermal motions in Rrp4 RNA-binding ring using thermal ellipsoid and B-factor analyses. (*a*) Overall analysis of the trimeric cap. (*b*) According to the thermal ellipsoid analysis, the most thermal flexible Rrp4 subunit is Chain I, but not Chain F, which displays the largest rigidbody motion. TLS sensors were obtained from TLS refinement in Refmac5 [48] (see Methods section for details) and plotted using Raster3D [51].(*c*) B-factor comparison of the Rrp4 between our *S. solfataricus* exosome (left) and that Lorentzen *et al* previously reported [30] (right). B-factor coloring: blue, 30 and below; red, 100 and above.

Besides conformational heterogeneity caused by the hinge motion, the Rrp4 trimer also exhibits distinct temperature factor distributions, which reflect the RNA binding ring's inherent conformational flexibilities. TLS refinement followed by thermal ellipsoids analysis revealed unique thermal motions in Chain I copy of Rrp4 (Fig. 4a, b). Although Chain C and Chain I of Rrp4 adopt very similar conformations, Chain I displays considerably higher thermal motion than Chain C, manifested by a higher average temperature factor of 81 versus 61 (Fig. 4a, c), as well as wider thermal ellipsoids in Chain I (Fig. 4b). Each Rrp4 subunit has distinct temperature factor distributions within the polypeptide (Fig. 4a, c). The shape of the thermal ellipsoids revealed that Chain I wobbles to a larger extent on top of the catalytic ring, and in a concentric motion relative to the central RNA-processing chamber. All above observations indicate that the Rrp4 trimeric cap is intrinsically flexible, despite bound to the relatively rigid catalytic ring. The observed conformational heterogeneity agrees perfectly with the function of the RNA-binding ring of the exosome to accommodate diverse RNA substrates.

## Discussion

Previous structural studies of the archaeal exosome from three different species all reported perfect three-fold symmetric subunit arrangements within this RNA processing machine. An interesting observation from these studies was that the RNA-binding ring displays large conformational differences among different species. For example, the conformation of the *S. solfataricus* exosome RNA-binding ring differs dramatically from what was seen in the *A. fulgidus* structure mostly due to rigid body rearrangement of the KH, S1, and NT domain locations [31] (r.m.s.d. of the Cα atom alignment in the 750-aminoacid Rrp4 region is 5.2 Å). This either suggests a faster evolution rate within the RNA-binding part of the exosome, or hints at inherent conformational flexibilities in this region. Through careful analyses of the conformation and thermal motion of each subunit, we provide evidence to support the higher-flexibility hypothesis. We showed that each Rrp4 subunit within the *S. solfataricus* RNA-binding ring adopts a distinct conformation and thermal motion distribution pattern. As a result, the previously assumed three-fold symmetric subunit arrangement is broken. Interestingly, it was found that significantly higher conformational flexibility and thermal motions are present in the RNA-binding ring of the crystal structures of the exosome-related PNPases from mesophilic bacteria, *Escherichia coli* [29] and *Streptomyces antibioticus* [25]. The RNA-binding ring (occupying 23% of the entire PNPase) in *E. coli* PNPase is so flexible that it could not be successfully traced from the electron density map [29]. It is therefore likely the RNA-binding ring may become even more dynamic in its native thermophilic environment.

What is the significance for maintaining the evolutionarily conserved conformation flexibility in the RNA-binding ring of the archaeal exosome and PNPase? We speculate two functional roles: (1) The conformational flexibility may play a passive role in accommodating the binding of diverse RNA substrates before threading the RNA into the catalytic ring. The fact that each of the three Rrp4 subunits possesses distinct thermal and conformational characteristics suggests that the exosome might respond to the substrate by activating one of the three Rrp4 subunits. It is also possible that the substrate binding is a cooperative event that involves more than one Rrp4 subunit. (2) On the other hand, the increased thermal motion and flexibility in this region may further play an active role in unzipping the secondary structures of the RNA substrates so that the RNA is ‘ready’ to pass through the narrow neck region of the exosome, which can only accommodate a single-stranded RNA. The RNA-binding ring therefore resembles an RNA helicase in its RNA-unwinding function. Consistent with this hypothesis, we observed that a strong tertiary structure, such as the HDV ribozyme, initially stalls the action of the *S. solfataricus* exosome, but can later be gradually degraded by the exosome, albeit at much slower rate (Fig. 1b).

## Materials and Methods

### Expression, and Purification of the 4-Subunit *Sulfolobus solfataricus* Exosome

Genes encoding Rrp4, Rrp41, Rrp42, and Csl4 have been cloned from the genomic DNA of the archaeal hyperthermophile *Sulfolobus solfataricus*. We designed a polycistronic construct to promote exosome assembly by co-expressing four subunits in *E. coli* simultaneously. This was inspired by the observation that the three Rrp genes are arranged as a superoperon (in the order of Rrp4-Rrp41-Rrp42) in the archaeal genomes [45]. The order of the genes was preserved in the polycistronic vector construction, followed by the insertion of the Csl4 gene [39], [41]. A His_6_-tag and a Tobacco Etch Virus (TEV) protease cleavage site were engineered into the N-terminus of Rrp4 to facilitate purification.

After overexpression in *E.coli*, the supernatant of the cell lysate was incubated at 75° for half an hour and precipitated using centrifugation to efficiently denature and remove *E. coli* proteins. Target proteins were then purified using nickel affinity chromatography using supernatant from the heat treatment. All four exosome subunits were present in the eluate in roughly predicted stoichiometry. Purified proteins were then dialyzed to a buffer containing 25 mM Tris-HCl pH 8.0 and 100 mM NaCl, incubated with TEV protease to remove the His_6_-tag, and further purified to homogeneity on an anion exchange chromatography (Mono Q column). Alternatively, the complex can by purified using size exclusion chromatography (Suprose 6 column). We confirmed the identity of the four archaeal proteins in the purified exosome by separation on SDS polyacrylamide gel (SDS-PAGE), followed by in-gel protease digestion, electrospray mass spectrometry, and data base matching (data not shown).

### RNase Assay

The 254-nt RNA substrate containing the hepatitis delta virus ribozyme followed by a 161-nt 3′-tail was generated from *in vitro* T7 RNA polymerase transcription reaction and purified following standard protocols as previously described [46]. The RNA was incubated with the purified archaeal exosome was incubated at 37°C under multiple turnover conditions (∼10 pmol enzyme, ∼40 pmol substrate) in a buffer containing 25 mM Tris-HCl pH 7.5, 100 mM NaCl, and10 mM inorganic phosphate (PO_4_^3−^). Control experiments were carried out in the same buffer in the absence of PO_4_^3−^ or in the presence of 20 mM SO_4_^3−^.

### Crystallization and Structure Determination of the Rrp4-Exosome

Our Rrp4-exosome isoform was concentrated to ∼10 mg/mL in a buffer containing 100 mM Tris-HCl pH 8.6, 200 mM Mg(Ac)_2_, and 25 mM (NH_4_)_2_SO_4_), and crystallized by hanging vapor diffusion against a well solution containing 30% PEG4000 with 100 mM Tris-HCl pH 8.6, 200 mM Mg(Ac)_2_. Crystals appeared after two months incubation at room temperature among heavy precipitations. The crystals were soaked in the well solution plus 10 mM NaSO_4_ for 10 minutes before flash-frozen in liquid nitrogen. A complete data set to 2.9 Å was collected at 90K from CHESS beamline A1. The crystal belongs to C2 space group with unit cell dimensions of 151 Å×145 Å×97 Å, and γ = 93.8°, as indexed and scaled by HKL2000 [42]. The apo Rrp4-exosome structure was solved by molecular replacement using PHASER [47] from the structure of the catalytic core of the *S. solfataricus* exosome (PDB code: 2BR2) [34] as the search model. Initial search using the nine-subunit complex resulted in very poor and untraceable maps in seven out of the nine subunits, while searching attempt with three copies each of individual Rrp41or Rrp42 subunits resulted in more than 100 steric clashes. Finally, a search using three pairs of Rrp41–42 heterodimer produced a reasonable solution and electrondensity map revealing traceable difference between the search model and our structure. Rigid body refinement by Refmac [48] further improved the overall electron density and revealed extra electron densities corresponding to Rrp4 in the F_o_-F_c_ difference map. Initially, strict three-fold non-crystallographic symmetry (NCS) was applied to refine the catalytic ring structure (Rrp41/42 heterotrimer) but was dropped when refinement can no longer improve the model and R_free_. Only two of the three Rrp4 subunits can be correctly placed by molecular replacement in PHASER using the published Rrp4 structure (PDB code 2JE6) [30], while attempting to position the third subunit resulted in broken densities. Therefore the third Rrp4 subunit was manually traced using COOT [49] and built from three- to ten-amino acid-long peptide segments of Rrp4. At this stage the strict NCS matrix was removed from subsequent refinements. Successive steps of manual refinement of all nine subunits using COOT [49] allowed tracing of more flexible regions as well as locating three sulfate ions at catalytic core domains., Multiple rounds of manual fitting followed by restrained and rigid body refinement in Refmac5 [48] reduced R_work_/R_free_ to 0.31/0.34 and positioned all three Rrp4 proteins into density map. At this stage the, R_work_/R_free_ stopped dropping due to over refinement in some of the subunits and under refinement in others. Therefore, we treated each of the nine subunits separately and independently subject them to energy minimization, simulated annealing, grouped B-factor refinement in CNS [50] while holding remaining eight subunits in position, which reduced R_work_/R_free_ to 0.29/0.31. We then defined a total of 15 translation-liberation-screw (TLS) groups in the entire structure: three of each Rrp41, Rrp42, N-ter, S1 and KH domain of Rrp4 subunit, to be used in TLS refinement in Refmac5 which ultimately reduced refinement statistics to the final R_work_/R_free_ = 0.268/0.289 (Table 1, Fig. 1d). The TLS refinement result is visualized by Raster3D [51]. The structure is verified by simulated annealing omit maps by excluding 7.5 percent of structure in each calculation.
